# The Updated Genome Warehouse: Enhancing Data Value, Security, and Usability to Address Data Expansion

**DOI:** 10.1093/gpbjnl/qzaf010

**Published:** 2025-02-20

**Authors:** Yingke Ma, Xuetong Zhao, Yaokai Jia, Zhenxian Han, Caixia Yu, Zhuojing Fan, Zhang Zhang, Jingfa Xiao, Wenming Zhao, Yiming Bao, Meili Chen

**Affiliations:** National Genomics Data Center, China National Center for Bioinformation, Beijing 100101, China; Beijing Institute of Genomics, Chinese Academy of Sciences, Beijing 100101, China; National Genomics Data Center, China National Center for Bioinformation, Beijing 100101, China; Beijing Institute of Genomics, Chinese Academy of Sciences, Beijing 100101, China; National Genomics Data Center, China National Center for Bioinformation, Beijing 100101, China; Beijing Institute of Genomics, Chinese Academy of Sciences, Beijing 100101, China; National Genomics Data Center, China National Center for Bioinformation, Beijing 100101, China; Beijing Institute of Genomics, Chinese Academy of Sciences, Beijing 100101, China; National Genomics Data Center, China National Center for Bioinformation, Beijing 100101, China; Beijing Institute of Genomics, Chinese Academy of Sciences, Beijing 100101, China; National Genomics Data Center, China National Center for Bioinformation, Beijing 100101, China; Beijing Institute of Genomics, Chinese Academy of Sciences, Beijing 100101, China; National Genomics Data Center, China National Center for Bioinformation, Beijing 100101, China; Beijing Institute of Genomics, Chinese Academy of Sciences, Beijing 100101, China; University of Chinese Academy of Sciences, Beijing 100049, China; National Genomics Data Center, China National Center for Bioinformation, Beijing 100101, China; Beijing Institute of Genomics, Chinese Academy of Sciences, Beijing 100101, China; University of Chinese Academy of Sciences, Beijing 100049, China; National Genomics Data Center, China National Center for Bioinformation, Beijing 100101, China; Beijing Institute of Genomics, Chinese Academy of Sciences, Beijing 100101, China; University of Chinese Academy of Sciences, Beijing 100049, China; National Genomics Data Center, China National Center for Bioinformation, Beijing 100101, China; Beijing Institute of Genomics, Chinese Academy of Sciences, Beijing 100101, China; University of Chinese Academy of Sciences, Beijing 100049, China; National Genomics Data Center, China National Center for Bioinformation, Beijing 100101, China; Beijing Institute of Genomics, Chinese Academy of Sciences, Beijing 100101, China; University of Chinese Academy of Sciences, Beijing 100049, China

**Keywords:** Genome assembly, Genome annotation, Genome database, Genome Warehouse, GenBank and RefSeq

## Abstract

The Genome Warehouse (GWH), accessible at https://ngdc.cncb.ac.cn/gwh, is an extensively-utilized public repository dedicated to the deposition, management, and sharing of genome assembly sequences, annotations, and metadata. This paper highlights noteworthy enhancements to the GWH since the 2021 version, emphasizing substantial advancements in web interfaces for data submission, database functionality updates, and resource integration. Key updates include the reannotation of released prokaryotic genomes, mirroring of genome resources from National Center for Biotechnology Information (NCBI) GenBank and Reference Sequence Database (RefSeq), integration of Poxviridae sequences, implementation of an online batch submission system, enhancements to the quality control system, advanced search capabilities, and the introduction of a controlled-access mechanism for human genome data. These improvements collectively enhance the ease and security of data submission and access as well as genome data value, thereby improving convenience and utility for researchers in the genomics field.

## Graphical Abstract

**Graphical Abstract:**
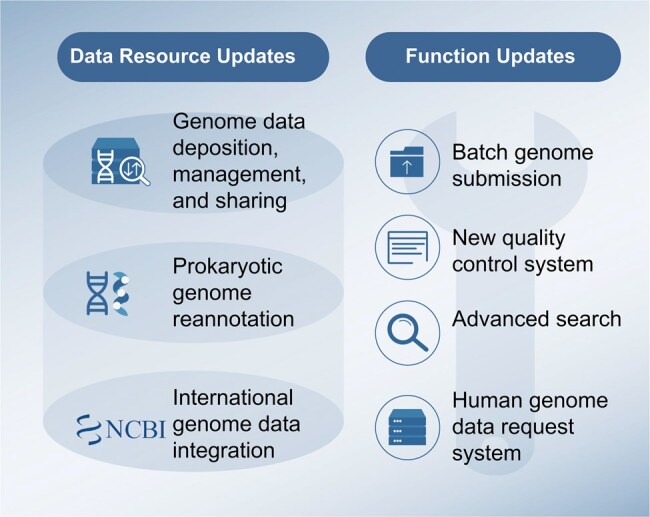

## Introduction

Genome Warehouse (GWH, https://ngdc.cncb.ac.cn/gwh) [1], one of the major resources in the National Genomics Data Center (NGDC, https://ngdc.cncb.ac.cn) [2] of China National Center for Bioinformation (CNCB, https://www.cncb.ac.cn) [3], serves as a publicly accessible repository of genome assembly data. Since its inception in 2017, GWH has been accepting genome assembly sequences, annotations, and metadata submissions from researchers worldwide. Compared to the well-known international genome databases such as National Center for Biotechnology Information (NCBI) Whole Genome Shotgun Submissions (WGS) [4] and European Bioinformatics Institute (EBI) Ensembl [5], which were established in the early 2000s with high data volume and significant influence, GWH’s data resources and service system are on relatively smaller scale and less efficient, but continuously improving and gradually catching up. It has been widely recognized by the scientific community, as testified by increasing users, downloads, and supported journals. As of Feb. 21, 2025, GWH has accepted 90,088 genome assemblies submitted by 1181 submitters from 363 institutions. Among these, 66,154 genome assemblies have been released and reported in 611 research articles in 164 scientific journals. However, the limited storage, network bandwidth, staff members, and funding remain limiting factors for GWH’s further development.

In the era of technology evolution and heightened user expectations for improved data services, GWH has encountered additional challenges. First, the tension between the burgeoning growth of genomic data and the efficiency of data submission is becoming increasingly prominent [6]. Second, despite the stable operation of the in-house quality control system, the growing complexity of data presents significant hurdles to the meticulous quality control and management of genomic data submissions. Third, since the genome annotations serve as the bridge between genome sequences and inferred biological functions, the omission of annotation significantly diminishes the potential value of genome data utilization and hinders the realization of the full spectrum of data-driven insights and discoveries. Finally, the inability to search and access the International Nucleotide Sequence Database Collaboration (INSDC) [7] data through GWH contributes to data fragmentation and the creation of data silos.

In addressing the challenges presented by the exponential expansion of data, the imperative of ensuring data quality, optimizing data utilization value, and facilitating streamlined data access for archival and management purposes, we introduce here an updated implementation of GWH. These enhancements mainly include the reannotation of prokaryotic genomes, mirroring of data from NCBI [4], introduction of an online batch submission pipeline, enhancements to data quality control, refined management of controlled-access to human genome data, and integration of advanced search functionalities. These improvements significantly elevate user experience and position GWH as a more efficient complement to the INSDC.

## Update overview and data statistics

This paper provides a comprehensive overview of these major developments. In terms of data resources, significant updates encompass prokaryotic genome reannotation and NCBI data integration. Concerning functionality, key updates include batch submission, quality control enhancements, advanced search capabilities, and the introduction of controlled-access systems. **Table 1** provides a detailed enumeration of all the updates.

**Table 1:** Comparison of GWH between the two versions in 2025 and 2021

Category	2025	2021
Prokaryotic genome reannotation	Available	/
NCBI genome assembly resource	NCBI GenBank and RefSeq genome assemblies	/
Poxvirus sequence resource	Available	/
Data submission	Online batch submission	Online single submission and offline batch submission
Accepted genome data	Full genomes of eukaryotes and prokaryotes as well as metagenomes	Assembled genomes of viruses, eukaryotes, prokaryotes, organelles, and plasmids as well as metagenomes
Quality control	NCBI table2asn and updated in-house script	In-house script
Search function	BIG Search and advanced search	BIG Search
Controlled access	Controlled human genome data can be downloaded upon request	/
Data download	FTP and HTTPS; online batch download	FTP
Language	English and Chinese	English
BLAST	Available	/
Data statistics	11 modules	8 modules

*Note*: GWH, Genome Warehouse; NCBI, National Center for Biotechnology Information; RefSeq, Reference Sequence Database; BIG, Beijing Institute of Genomics; FTP, File Transfer Protocol; HTTPS, Hypertext Transfer Protocol Secure; BLAST, Basic Local Alignment Search Tool.

**Figure 1:**
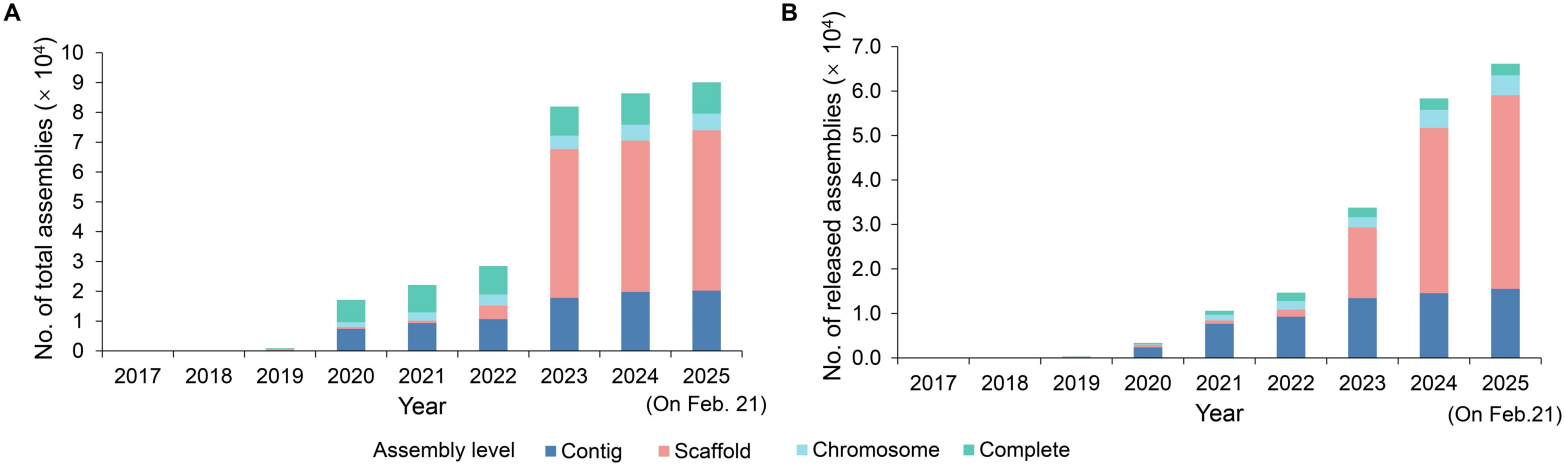
**Statistics of directly submitted genome assembly data in GWH** **A**. Statistics of total directly submitted genome assembly data. **B**. Statistics of publicly released genome assembly data. These statistical data are as of Feb. 21, 2025. GWH, Genome Warehouse.

As of Feb. 21, 2025, the submission volume of GWH has shown remarkable growth, with a total of 90,088 genome assembly submissions accepted. These submissions contain 5547 organisms from a diverse community of 1181 submitters, representing 363 organizations. Notably, 24 international submitters from 5 countries have contributed to this global initiative. GWH has successfully released 66,154 genome assemblies, representing 4384 distinct organisms across various assembly levels (**Figure 1**). The released genome assemblies span diverse divisions, including animals (3095), plants (3722), fungi (402), protists (951), bacteria (7620), archaea (130), viruses (2454), metagenomes (44,184), and others (3596). These valuable genomic resources have been disseminated through 611 articles published in 164 journals.

Moreover, GWH has received 53 access requests for controlled human genome data, with 19 of them having been authorized by the data provider. The GWH website has garnered attention from a global audience, attracting 233,628 unique users from 171 countries/regions. From 2021 to 2025, the daily download of released genome assemblies consistently surpassed 500 times. Recognizing its dedication to ongoing maintenance and updates, GWH has received certifications from FAIRsharing.org and re3data.org, validating its adherence to standards for findability, accessibility, interoperability, and reusability. In summary, these diverse statistical metrics collectively illustrate the substantial growth of GWH, emphasizing its significant potential to support global research efforts in genomics.

## Data resource updates

### Prokaryotic genome reannotation

**Figure 2:**
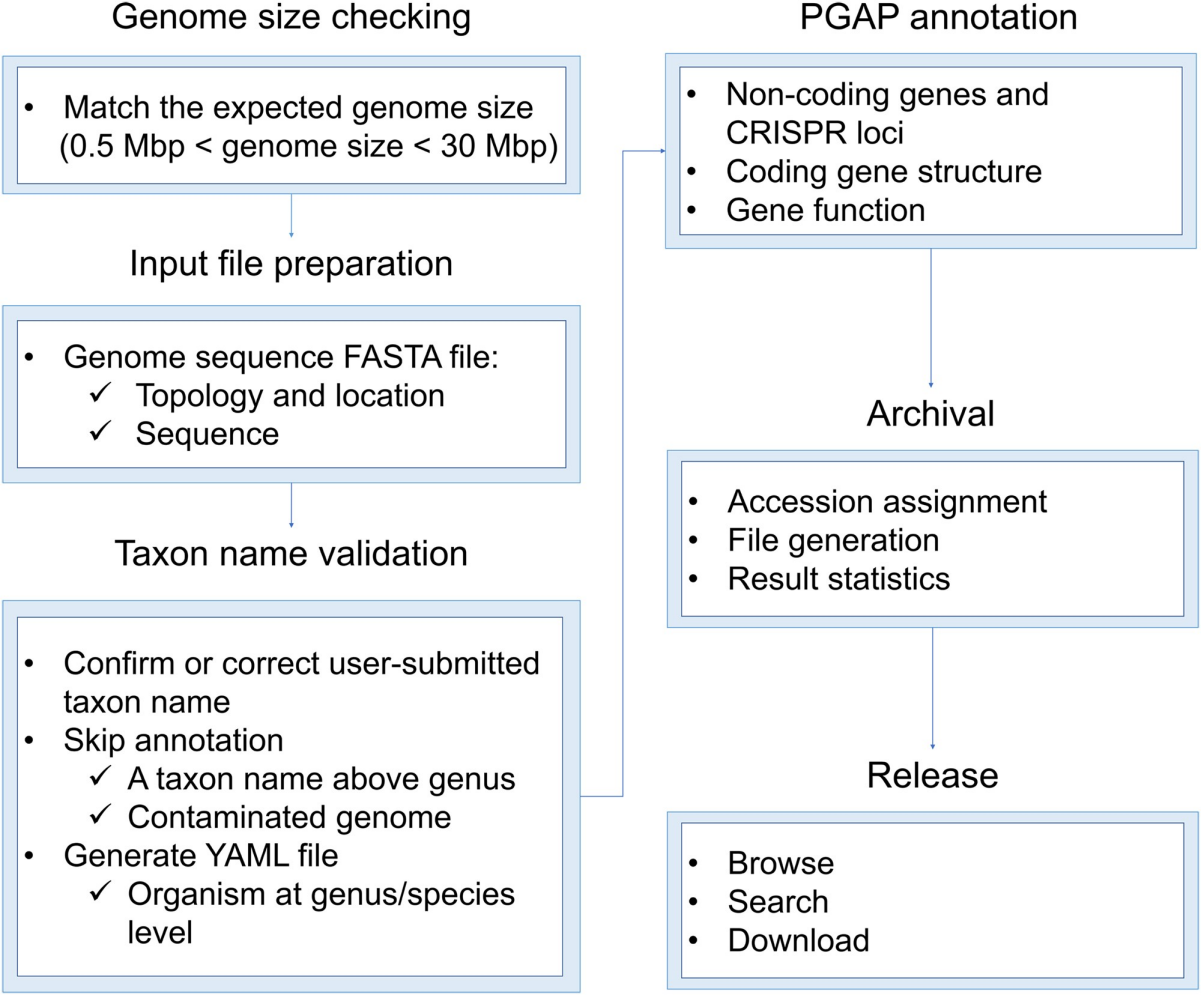
**Workflow of prokaryotic genome reannotation in GWH**The process of prokaryotic genome reannotation in GWH involves preprocessing and annotation of genome sequences, annotation result archival, and release of annotation results. PGAP, Prokaryotic Genome Annotation Pipeline; FASTA, FAST-All; YAML, Yet Another Markup Language; CRISPR, clustered regularly interspaced short palindromic repeats; Mbp, mega-base pairs.

Among the 14,637 prokaryotic genome submissions to GWH, less than 0.5% are accompanied by genome annotations. Moreover, the submitted genome annotations primarily contain gene structure predictions, lacking comprehensive gene function annotations. To enhance the utility of released prokaryotic genomes for associated research endeavors, this version of GWH introduces an automatic reannotation pipeline utilizing the Prokaryotic Genome Annotation Pipeline (PGAP) [8] from NCBI [4]. This initiative aims to provide standardized and unified genome reannotations, thereby facilitating progress in functional genomics research. The reannotation pipeline involves the following six processes (**Figure 2**). (1) Genome size checking: genomes matching the expected genome size range [0.5 mega-base pairs (Mbp) < genome size < 30 Mbp] are retained. (2) Input file preparation: topology and location information (affecting genetic codon selection) in genome sequence files are integrated into the definition line if known. (3) Taxon name validation: the taxon name is crucial as it influences genetic codes and reference protein selection. To ensure completeness and precision, the automatic pipeline incorporates a PGAP taxon validation, which confirms or corrects the taxon name based on the average nucleotide identity (ANI) between the query genome and the type genomes stored in GenBank [9]. If misassigned check result occurs, the taxon name is corrected to align with the best-matched taxon name before PGAP annotation. If contamination is identified, the reannotation procedure is terminated. Additionally, PGAP requires the submitted taxon rank to be at least at the genus level; hence, a hypothetical taxon name is employed for taxon correction if the submitted taxon is above genus. Only genomes with confirmed results or those with misassigned results exhibiting a high confidence level undergo PGAP annotation. (4) PGAP annotation: the annotation results contain structural ribosomal RNAs (rRNAs), transfer RNAs (tRNAs), small non-coding RNAs (ncRNAs), repetitive regions, protein-coding genes, and protein names. (5) Archival: upon completion of PGAP, the pipeline assigns unique accession numbers, generates downloadable files, and implements comprehensive backup measures. (6) Release: subsequently, the reannotation results are released and displayed on the page of original submitted genome sequences as associated data pairs, readily browsable, retrievable, and downloadable. These files are publicly accessible at https://download.cncb.ac.cn/gwh/Reannotation. As of Feb. 21, 2025, GWH has completed annotation for 6880 out of 7750 released prokaryotic genomes. The remaining genomes exhibit abnormal sizes (115), lack taxonomy rank at genus/species levels and cannot be corrected (636), or contain contamination (119).

### NCBI data integration

To enhance data sharing and ensure comprehensive accessibility to all genome assembly-related information, GWH has implemented an in-house mirroring pipeline. This pipeline mirrors the entire genome assembly data from GenBank [9] and Reference Sequence Database (RefSeq) [10], capturing metadata primarily through NCBI Entrez Programming Utilities (E-utilities), along with genome sequences, annotation files, and corresponding statistical data sourced from NCBI File Transfer Protocol (FTP) files (https://ftp.ncbi.nlm.nih.gov/genomes/genbank/ and https://ftp.ncbi.nlm.nih.gov/genomes/refseq/). Additionally, GWH retrieves taxon information from the NCBI Taxonomy Database, including scientific names, common names, synonymous names, and taxonomy lineages. This extensive dataset seamlessly integrates with data directly submitted to GWH. The platform ensures continuous synchronization, updates, and comprehensive support for data searching, browsing, and downloading (https://download.cncb.ac.cn/assembly/ncbi/). As of Feb. 21, 2025, GWH has successfully mirrored approximately 2.54 million GenBank and 0.48 million RefSeq genome assembly records, along with associated data files. The all-in-one integration of genome data enhances researchers’ access to NCBI data (https://ngdc.cncb.ac.cn/gwh/browse/assembly?source=ncbi).

In our effort to integrate virus genome assembly data, we aggregate genomic and protein sequences, along with associated metadata related to the Poxviridae family sourced from NCBI, to establish the poxvirus module (https://ngdc.cncb.ac.cn/gwh/browse/virus/poxviridae) [11]. The resource undergoes daily updates by an in-house script, ensuring timely information availability. Users are able to browse and filter sequences based on diverse criteria, facilitating efficient data exploration and retrieval.

## Function updates

### Data submission and quality control

To enhance user experience with a focus on friendliness, convenience, and efficiency in data submission, the latest version of GWH introduces an online batch submission functionality. This feature empowers submitters to submit all genome assembly data associated with the same BioProject and publication simultaneously. Users can provide unique metadata for each assembly, such as BioSample accession, assembly name, assembly level, and genome sequence filename, using an Excel template file. Following the submission of metadata and data files, the entire batch undergoes a comprehensive quality control and feedback process. This streamlined approach eliminates the need for repetitive submission of common metadata, significantly reducing the submission process duration and markedly enhancing submission efficiency.

High-quality genomic data form a crucial foundation for accurate and reliable downstream omics data analysis. Recognizing this, GWH has undertaken a comprehensive update to its quality control system to ensure the quality of submitted data. These enhancements consist of four key components. (1) Genome size check: GWH cross-references genome size data with information from NCBI (ftp://ftp.ncbi.nlm.nih.gov/genomes/ASSEMBLY_REPORTS/species_genome_size.txt.gz) to verify a reasonable genome size. (2) Lineage rank check: GWH requires a genus/species-level description for each assembly, ensuring taxonomic clarity. (3) Enhanced precision in genome annotation validation: building upon the original quality control system, GWH integrates NCBI’s table2asn tool (https://www.ncbi.nlm.nih.gov/genbank/table2asn/) to validate genome annotations using stricter criteria for gene structure and function annotations, as well as for the file format, completeness, and consistency of gene structures. (4) Warnings: in addition to identifying fatal errors, GWH now issues warnings, allowing submitters to consider modifications based on biological context of their data. With over 800 different types of error/warning messages, GWH expands its quality control capabilities to ensure rigorous scrutiny of genome annotation content.

Furthermore, GWH has refined the accepted data types, placing particular emphasis on comprehensive eukaryotic and prokaryotic genomes, as well as metagenomes. Given the rapid expansion of metagenomics data, which represents the collective genomes of microorganisms within a specific environment, the availability of rich metadata and accurate genomic data is essential for facilitating scientific exploration of microbial community composition and function. To support this endeavor, GWH has enhanced its data organization structure and substantially augmented the collection of metadata specifically related to metagenome-assembled genomes (MAGs). This update ensures seamless linkage among sequencing raw data, primary metagenome assembly, binned metagenome, and MAGs. Furthermore, this version incorporates essential metadata pertaining to binning analysis, quality assessment, and taxonomic identification for both binned metagenomes and MAGs, such as completeness, contamination rate, binning analysis software, and associated parameters. By including rich metadata and a well-organized data structure, GWH allows users to select and utilize metagenome-associated data more efficiently, thereby enhancing the scientific exploration of microbial communities.

As sequencing technology advances, an increasing number of new organisms undergo sequencing and assembly. However, the taxonomy information for these species may not be readily available in the NCBI Taxonomy Database, hindering the sharing process for their genomes. To address this challenge, GWH allows users to contribute the pertinent taxonomy information of newly sequenced organisms with supporting evidence from publications or other databases such as Genome Taxonomy Database (GTDB) [12]. Currently, GWH has incorporated data for 1542 new organisms, comprising 1783 genome assemblies. Furthermore, the genetic code determines the codon usage for each coding sequence (CDS) within an organism. Notably, some GWH submitters have identified inaccuracies in the genetic code for lesser-known organisms in NCBI’s Taxonomy Database and have provided corrected genetic code values following thorough analysis. Warning messages about these exceptions are prominently displayed on the detailed web page for each assembly. GWH currently features 9 organisms across 18 genome assemblies, employing these updated genetic codes. These data primarily stem from the genomes of protists contributed by the Protist 10,000 Genomes (P10K) project [13], offering insights into substantial genetic code variations among ciliates.

### Data retrieval, search, and access

The latest version of GWH introduces significant enhancements to data retrieval, download, and access. GWH now provides personalized data retrieval services through the introduction of an advanced filtering function on the assembly browse page. Users can tailor their searches by setting single or multiple filter conditions, such as scientific name, reannotation status, and GC content range, enabling convenient identification and retrieval of assemblies of interest. To enhance the convenience of data download for GWH users, we have introduced a batch download function for filtered results. This feature is applicable to both GWH assemblies/reannotations and integrated NCBI assemblies/reannotations. Additionally, GWH has incorporated the Hypertext Transfer Protocol Secure (HTTPS) download method to adapt to the evolving landscape of web browsers. These updates are designed to improve the efficiency and user-friendliness of data retrieval and download services within GWH.

Furthermore, GWH has introduced an advanced search system with 18 conditions (*e.g.*, BioProject, BioSample, assembly level, and organism group) for all released genome assemblies, including those integrated from GenBank and RefSeq. This robust system supports both single-condition and multi-condition queries, allowing users to specify conditions for particular fields or across all fields. Importantly, users can retrieve genomes of corresponding organisms from any rank in the lineage, adding flexibility to their search conditions. The browsing session retains a search history, enabling users to incorporate each historical search as a condition in the search expression. On the result page, entries are categorized and aggregated, with additional multi-dimensional filtering options provided to further customize search results. Users can select metadata and data download links of interest from the search results, and download them as a text file. Alternatively, a batch download function is provided for downloading the metadata and data files of resulting genome assemblies to improve convenience for GWH users. The advanced search function empowers users to construct detailed search formulas, facilitating precise data searches and ensuring that the results are concise and accurate. This enhancement greatly enhances the usability and efficiency of the search functionality within GWH.

To meet the unique management needs of human genetic resources, we have implemented a controlled-access data request and authorization management system. Access to controlled data is restricted to users who have obtained explicit permission from the data owner, and the downloaded data can only be used within its designated validity period. Furthermore, users are obligated to adhere to legal and compliance standards, conducting research solely within the declared scope. This system supports online interactive operations and ensures prompt email notifications, enabling efficient communications between applicants and data owners throughout the application and processing stages. It fosters efficient collaboration and contributes to the protection of sensitive information and responsible utilization of controlled data.

## Future directions

GWH serves as an accessible and user-friendly platform dedicated to archiving, searching, and sharing genome data, complemented by a range of associated services. However, the accelerating influx of genome data into GWH presents significant challenges in processing and managing this vast dataset. Therefore, GWH remains committed to the continuous refinement of its data curation platform, with a focus on enhancing automation and intelligence across all processes. As genomic research progresses, GWH aims to broaden its accepted data types and reinforce quality control measures, including the incorporation of pangenome data in Graphical Fragment Assembly (GFA) format. Additionally, GWH plans to explore the integration of eukaryotic genome annotation services to enhance the value of submitted genome data and promote advancements in related research fields. This initiative includes providing comprehensive gene function annotations, Gene Ontology (GO) functions, metabolic pathways, and protein domains. GWH will utilize the open-source NCBI Eukaryotic Genome Annotation Pipeline - External (EGAPx, https://github.com/ncbi/egapx), the pipeline used by RefSeq, to establish its eukaryotic reannotation pipeline for supported organisms. For other organisms, GWH will employ an optimized pipeline developed in collaboration with our partner institute to ensure comprehensive genome reannotations. Looking ahead, GWH envisions facilitating the sharing and exchange of all publicly available genome assembly data with INSDC members. By doing so, GWH seeks to contribute to a global collaborative effort and provide researchers worldwide with comprehensive and enriched genomic data resources, thereby advancing scientific discovery in genomics.

## Data Availability

GWH is freely accessible at the NGDC, CNCB (https://ngdc.cncb.ac.cn/gwh/).

## CRediT author statement

**Yingke Ma:** Software, Writing – original draft, Writing – review & editing. **Xuetong Zhao:** Investigation, Methodology, Data curation, Writing – original draft. **Yaokai Jia:** Software, Writing – original draft. **Zhenxian Han:** Software. **Caixia Yu:** Data curation. **Zhuojing Fan:** Methodology. **Zhang Zhang:** Writing – review & editing. **Jingfa Xiao:** Writing – review & editing. **Wenming Zhao:** Writing – review & editing. **Yiming Bao:** Conceptualization, Writing – review & editing, Supervision. **Meili Chen:** Project administration, Investigation, Methodology, Data curation, Writing – original draft, Writing – review & editing. All authors have read and approved the final manuscript.
